# Assessing parental self-efficacy for obesity prevention related behaviors

**DOI:** 10.1186/1479-5868-11-53

**Published:** 2014-04-22

**Authors:** Julie A Wright, William G Adams, Robert G Laforge, Donna Berry, Robert H Friedman

**Affiliations:** 1 Department of Exercise and Health Sciences, UMass Boston, 100 Morrissey Blvd, Boston, MA 02125, USA; 2 Department of General Pediatrics, Boston University School of Medicine, 88 East Newton Street, Boston, MA 02118, USA; 3 Department of Psychology, University of Rhode Island, Kingston, RI 02818, USA; 4 Dana-Farber Cancer Institute, 450 Brookline Avenue, Boston, MA 02215, USA; 5 Section of General Internal Medicine, Boston University School of Medicine, 801 Mass Ave, Boston, MA 02118, USA

**Keywords:** Obesity prevention, Self-efficacy, Scale development, Pediatric primary care, Parents

## Abstract

**Background:**

Reliable, valid and theoretically consistent measures that assess a parent’s self-efficacy for helping a child with obesity prevention behaviors are lacking.

**Objectives:**

To develop measures of parental self-efficacy for four behaviors: 1) helping their child get at least 60 minutes of moderate intensity physical activity every day, 2) helping one’s child consume five servings of fruits and vegetables each day, 3) limiting sugary drinks to once a week, and 4) limiting consumption of fruit juice to 6 ounces every day.

**Methods:**

Sequential methods of scale development were used. An item pool was generated based on theory and qualitative interviews, and reviewed by content experts. Scales were administered to parents or legal guardians of children 4–10 years old. The item pool was reduced using principal component analysis. Confirmatory factor analysis tested the resulting models in a separate sample.

**Subjects:**

304 parents, majority were women (88%), low-income (61%) and single parents (61%). Ethnic distribution was 40% Black and 37% white.

**Results:**

All scales had excellent fit indices: Comparative fit index > .98 and chi-squares (*Pediatrics* 120 Suppl 4:S229-253, 2007) = .85 – 7.82. Alphas and one-week test-retest ICC’s were ≥ .80. Significant correlations between self-efficacy scale scores and their corresponding behaviors ranged from .13-.29 (all p < .03).

**Conclusions:**

We developed four, four-item self-efficacy scales with excellent psychometric properties and construct validity using diverse samples of parents.

**Trial registration:**

Clinical trial registration: NCT01768533.

## Background

Pediatric health guidelines recommend multiple lifestyle behaviors to prevent childhood obesity [1-4]. While there is evidence for the specific behaviors to target, there is less evidence about how to effectively change them. Targeting parents as the agents of change holds promise given their significant role in children’s diets and physical activity [5-8] and their role in providing the social and environmental support for the multiple health behaviors involved in managing a child’s healthy weight. There is evidence to suggest that targeting parents exclusively has been an effective method in the treatment of childhood overweight and obesity [9,10].

Given the parents’ role in health weight management, interventions and treatment plans should include and assist parents in the behavior change process. A strong determinant of health behavior change is self-efficacy [11,12], a construct from social cognitive theory defined as one’s confidence in his or her ability to engage in the target behavior under a range of difficult situations [13]. Studies suggest that parent self-efficacy is important to childhood obesity [9,14,15], levels of physical activity [16] and consumption of fruits and vegetables [17]. To better understand the parent’s role in childhood weight management and parent self-efficacy, reliable, valid and theoretically consistent measures of parent self-efficacy are needed.

Several studies have developed measures for parental self-efficacy for behaviors typically targeted in obesity prevention or treatment, but there is limited support for their use. There was only one study [17] that has adequately defined the construct of self-efficacy, used well accepted methods of scale development, and reported good psychometric properties. The majority of scales query self-efficacy with one item (question) [14,15,18,19] which may not be enough to adequately define a construct that is stable enough to use in future studies [20,21]. Taveras et al. [15] developed a parental confidence questionnaire for use in the clinical setting that included one item per behavior. Parents of overweight children 2–12 years old were asked about their confidence in changing family’s eating and activity patterns, limiting TV, removing TV from bedroom, reducing soda, juice and other sweet drinks and reducing fast-food. West and Sanders [14] created a problem checklist for parents of obese children 4–11 years old and asked parents to rate their confidence in managing specific behaviors (e.g., confidence in managing how quickly a child eats or how much TV is viewed). Neither of these scales assessed self-efficacy in the context of specific barriers.

More recently, Nelson & Davis [22], developed a multi-behavior, 41-item parent efficacy for child health weight behavior scale using Bandura’s conceptualization of self-efficacy for minimizing fat and sugar intake, eating healthy snacks, engaging in no more than two hours of sedentary activity a day, engaging in one hour of physical activity, eating at least three servings of fruit and eating four or more servings of vegetables. There are caveats with this scale that include a large number of items per behavior and questionable methodology (e.g., the same sample of participants was used for the exploratory and confirmatory phase of the scale’s development).

The scale with the most validity to date was developed using stronger methodology by Cullen et al. [17], a 20-item parent self-efficacy instrument for providing and encouraging fruit and vegetable (F&V) intake in 4^th^ to 6^th^ grade children. The items reflected parental self-efficacy for modeling the consumption of F&V, planning and encouraging F&V consumption, and making F&V available. One limitations is its length (20 items) that possibly limits its usability, especially in studies that involve multiple behavioral measures.

The primary purpose of this study was to develop and evaluate reliable and valid scales that assess self-efficacy for multiple behaviors associated with childhood obesity prevention. A secondary aim was to develop shorter scales for researchers who study multiple behaviors, thus reducing respondent burden, and for pediatric clinicians who assess parental confidence. Based on previous research, it was expected that a one factor model would fit the data [23-25] with four to five items adequately defining the latent variable. The second hypothesis was that the behavioral levels for each of the four target behaviors would increase as self-efficacy increased, providing support for construct validity [26].

## Methods

### Sample

Participants were recruited through advertisements posted in local newspapers and the miscellaneous section of Boston Craig’s list and flyers posted in health and community centers in the city of Boston. Inclusion criteria were: self-identifying as the principal care giver of a child between the ages of 4–10 years old, attendance at the last two of the child’s well-child annual exams, and willingness to bring the child to the study office for weight and height measurements. These inclusion criteria were used to capture parents, grandparents and legal guardians [herein called parents] who were more likely to be responsible for the child’s diet, physical activity and healthcare decisions. Informed written consent was obtained from the parent and verbal assent was obtained from the children at the study office. The study was approved by the institutional review boards at Boston University and University of Massachusetts Boston.

### Procedures

The study protocol was divided into three study office visits. At the first visit, a trained research assistant measured the height and weight of the child and parent and administered the Rapid Estimate of Adult Literacy in Medicine [27] to the parent. Height and weight were measured without shoes using a portable stadiometer (Seca model 214) and scale (Seca model 882). At the second visit, psycho-social and behavioral surveys were administered. Parents were invited to return to the study office to complete the paper-pencil surveys a second time. A window of 6–8 days was given for the parent to return; however, after 100 parents completed the one-week test re-test visit, the remaining parents were invited to return 12–14 days after the first visit to assess a longer time period for test retest. All surveys were self-administered, paper-pencil and completed by the parent. Parents were also given the option to combine the first two study office visits for convenience. Parents received gift card incentives for completing each visit.

### Development of the scales

The primary aim of this survey study was to develop reliable and valid measures of parental self-efficacy for four behaviors: 1) helping their child get at least 60 minutes of moderate intensity physical activity every day, 2) helping one’s child consume five servings of fruit and vegetables each day, 3) limiting sugary drinks to once a week, and 4) limiting consumption of fruit juice to 6 ounces daily. These four behaviors were selected because they were identified by major child health care professional organizations (e.g., American Academy of Pediatrics, American Heart Association, American Medical Association, Centers for Disease Control and Prevention) to encourage primary care providers to address them during primary care visits as part of health maintenance and obesity prevention efforts [1,4]. Targeting these four behaviors is consistent with previous pediatric health promotion efforts such as the 5-2-1-0 catch phrase (i.e., eat five servings of fruits and vegetables, two hours or less of TV or screen-time, one hour of moderate intensity physical activity, and no sugar-sweetened beverages) [28,29].

The items (e.g., the statements) for the four survey scales were selected in a multistep process. A review of the literature was used to collect any existing measures that would inform item creation. A set of 15–20 items were generated for each of the four behaviors and circulated to six experts from the disciplines of childhood overweight/obesity, health communication, exercise, nutritional sciences, and survey development. Experts were asked to evaluate how well the items represented the construct of SE. Items were added and refined based on their feedback. The resulting surveys were completed by six parents who met the inclusion criteria described previously. The parents were interviewed about the meaning of the items and feedback was used to improve the clarity of the wording and comprehension for a range of literacy levels.

#### Self-efficacy scales

For this study, self-efficacy was defined as confidence in one’s ability to perform a criterion behavior in a variety of difficult situations. According to Bandura, items should represent gradations of challenges to adequately represent this type of perceived self-efficacy [13,26]. Additionally, multiple items are necessary to adequately represent a latent construct.

All of the self-efficacy scales used the same stem, “how sure are you that you could,” which was followed by the criteria for the target behavior: 1) How sure you are that you could help your child get 1 hour of moderate intensity physical activity, 2) How sure are you that you could provide your child with 5 servings of fruits and vegetables everyday in the following situations? 3) How sure you are that you could limit your child to 1 serving per week of sugary drinks in the following situations? 4) How sure are you that you could limit your child’s juice intake to no more than 6 ounces of 100% fruit pure juice each day in the following situations? Twenty-one difficult situations were queried for each target behavior except for fruits and vegetables which had 18 situations. A 5-point Likert scale (1 = not sure; 2 = a little sure; 3 = sure; 4 = very sure; 5 = extremely sure) was used.

#### Measures of health behaviors

Behavioral levels of sugary drinks, fruit juice, fruit and vegetable consumption and physical activity were assessed with parent reported paper-pencil questionnaires, i.e., proxy reported levels of the child’s behavior. Dietary variables were assessed with the Block Dietary Data Systems Kids Food Screener version 2 which asks about the number of days in the last week each of 39 foods were consumed by the child and how much in one day with a set choice of three possible portion sizes (e.g., a little, some, a lot) [30-32]. The Food Screener uses this information to generate the child’s consumption of cup equivalents for fruits and vegetables, grams of sugar derived from sweetened beverages, and the frequency of consumption of sugary beverages. A separate paper-pencil questionnaire asks parents to report amounts of regular soda, fruit juice, fruit drink and water consumed on a typical day. The response options for regular soda were none, 12 ounces (1 can), 16 ounces (1 bottle), 24 ounces (2 cans), 32 ounces (2 bottles), more than 32 ounces (more than 2 bottles) while the response options for 100% pure fruit juice with no added sugar, and fruit drink (not 100% juice) were none, less than 6 ounces (less than 1 juice box), about 6 ounces (1 juice box), about 12 ounces (2 juice boxes), about 24 ounces (3 juice boxes), more than 24 ounces (4 juice boxes). Categories for water were none, less than 8 ounces (less than 1 glass), about 8 ounces (1 glass), about 16 ounces (2 glasses), about 24 ounces (3 glasses), and more than 24 ounces (more than 3 glasses). Children’s level of physical activity was assessed with the Amherst questionnaire [33]. This questionnaire asks parents to report on whether their child participated in a list of 20 different physical activities in the past seven days, the number of days each activity was done and minutes each time. Metabolic equivalents were assigned to each of the 20 physical activities on the questionnaire using the compendium of physical activities [34]. Parents were also asked two open ended question to assess outdoor time on a typical weekday and weekend, e.g., “how much time would you say your child spends playing outdoors on a typical day?”

### Data analysis

Split-half cross validation procedures were used for each of the four scales developed which included randomly splitting the sample into an exploratory and confirmatory halves, using the exploratory half of the sample to reduce the item pool, and using the confirmatory half of the sample to confirm that the set of items produces a good fitting model [35]. The sample was split into two halves (exploratory and confirmatory) using the random selection procedure in SPSS version 17.0 (SPSS, Chicago, IL). Missing data were examined for each sample. If less than ten percent of the items within a case were missing, those items were replaced with the sample’s mean for that specific item. This happened in less than two percent of the cases. Participants who were missing more than ten percent of the items for a particular scale were not included in the development of that scale.

#### Exploratory phase

Descriptive statistics were used to examine the normality and quality of the items. A quality item should have a mean that represents the middle of the response scale and a larger standard deviation. Items with means at the extreme high or low ends of the scale, smaller standard deviations and large negative or positive skewness or kurtosis can influence the reliability and validity of the scale, i.e., how well the scale defines the construct [21,35].

Item reduction was performed using Principal Component Analysis with varimax rotation with Kaiser normalization. The number of components was determined using a combination of three methods: parallel analysis [36], mean average partial (MAP) [37] and scree plots [21]. All of these methods were used to make a decision on the number of components. The decision to delete items was based on the item’s loading and on its depth and breadth of construct. Complex items or items with loadings less than 0.4 were deleted. The goal of item reduction was to achieve a scale of four items to reduce participant burden when completing a questionnaire that includes assessment of multiple behaviors yet maintain a robust and stable scale that adequately represents the construct of self-efficacy for each of the target behaviors [21]. When seven items remained, item-total statistics (Cronbach’s alphas, item means, variances, and correlations) were examined to further cull the items. Descriptive statistics, random selection of cases and principal component analysis were performed in SPSS version 17.0 (SPSS, Chicago, IL).

Exploratory factor analyses were run in EQS for Windows 6.1(Multivariate Software, Inc.) with five items using the maximum likelihood (ML) model. Multiple fit indices were used to examine model fit; Chi-square, Chi-square/degrees of freedom ratio, comparative fit index (CFI), root mean squared error of approximation (RMSEA), and standardized root mean residual (SRMR) [38-40]. Generally a good fitting model is one that has a non-significant Chi-square, a Chi-Square/degrees of freedom ratio < 2, CFI > .95, RMSEA < .05, SRMR < .10 [38,39]. Within the exploratory phase, alternate items were explored if the Chi-square was significant or RMSEA was above 0.10. If the five item model had a Chi square with a *p* > .05 and RMSEA < .10 then the feasibility of the four item model was explored. The best fitting model was selected for the confirmatory phase.

#### Confirmatory phase

The confirmatory factor analysis was performed in EQS using the same parameters and fit indices as in the exploratory phase.

#### Reliability

Internal consistency reliability was assessed using Cronbach’s alpha coefficient. Constructs stability was also examined using test-retest of the sum scores for each self-efficacy scale. One-way analysis of variance was performed to examine the differences between test, or repeatability. The R was computed using R = (MSS – MSE)/MSS. Participants who were missing one of the scale items were not included in the analysis. Fewer than six cases were removed across all analyses.

#### Validity

The construct validity of the scales was assessed by comparing parent-reported levels of their child’s behavior with the self-efficacy scales. Those parents who have higher self-efficacy scores should be reporting that their child has higher levels of the desired health behavior and lower levels of the unhealthy behavior. A linear relationship between behavioral level and self-efficacy score has been found previously [24,40]. Spearman rank correlations were used to assess construct validity between the scale scores and continuous behavioral variables. Analysis of variance (ANOVA) with Tukey post hoc pairwise comparisons were used to assess the levels of self-efficacy scores across response categories.

## Results

### Participants

Of the 319 parents who initially agreed to participate, 304 (95%) completed the questionnaires and 49% completed the questionnaire a second time for test-retest purposes. Weight and height were measured on 268 (88%) of the parents and 266 (87.5%) of the children. The participant sample was representative of a diverse urban population of parents in Massachusetts. The majority were overweight, female, not married with a total household income of less than 40,000 US dollars per year. No ethnic group was in the majority. Table 1 displays demographic characteristics of the analytic sample.

**Table 1:** Demographic characteristics of the sample used in the analysis

**Variable**	**Mean (SD) or n**		**Sample reporting**
	**(%)**		**(n)**
Parent age (yrs), M (SD)	37.3	(8.3)	254
Female, n (%)		(88%)	283
Not married (single), n (%)		(53%)	296
Income <40000 US dollars, n (%)		(60%)	295
Full or part-time employment, n (%)		(59%)	297
Education level			297
High school or less, n (%)		(23%)	
Some college, n (%)		(38%)	
College degree, n (%)		(39%)	
Hispanic/Latino origin (yes), n (%)		(21%)	297
Race			284
Black/African American, n (%)		(46%)	
White, n (%)		(39%)	
Mixed, n (%)		(19%)	
Parent BMI (kg/m^2^), M (SD)	30.4	(7.6)	268
Overweight^a^, n (%)		(27%)	
Obese^a^, n (%)		(46%)	
Child Age (yrs), M (SD)	6.6	(2.1)	266
Child BMI (kg/m^2^), M(SD)	18.2	(3.8)	
Overweight^b^ , n (%)		(22%)	
Obese^b^, n (%)		(21%)	

^a^Overweight for adults was defined as a BMI ≥25 and <30 and obese defined as BMI ≥30.

^b^Overweight for children was defined as having a BMI-for-age within the 85^th^%tile < 95^th^% tile and obese defined as BMI-for- age within the > =95th% tile [41].

The results of both the exploratory and confirmatory phase are summarized in Table 2. All scales were reduced to four items in the exploratory phase. Item loadings ranged from .73 to .89 and .60-.90 for the confirmatory phase. Cronbach’s alphas were above .80 for both phases indicating excellent interrelatedness among the items. The results of the confirmatory factor analyses are presented in Table 3. The models fit the data well overall. All scales except sugary drinks had a non-significant Chi-square and a Chi-Square/degrees of freedom ratio < 2, and all scales had a CFI > .95 and SRMR < .10. In contrast, the upper limits of the 90% CIs for the RMSEA were > .10 indicating a poor fit. Table 4 displays the test-retest results for one week and two week intervals. The average intraclass correlation (ICC) were significant at p < .009 and above .80, indicating excellent repeatability.

**Table 2:** Mean item scores and factor loadings from the exploratory principal components analysis and confirmatory factor analysis

**Item**	**Exploratory sample**				**Confirmatory sample**			
**Behavior**	**Loading**	**Mean**	**sd**	**Alpha**	**Loading**	**Mean**	**sd**	**Alpha**
Physical activity		N = 148		.85		N = 154		.80
When there are too many other things to worry about	.87	3.1	1.3		.71	2.8	1.2	
When money is tight	.85	3.2	1.3		.74	3.0	1.3	
When you do not like to exercise or play	.82	3.4	1.3		.76	3.2	1.3	
When your child is tired	.78	2.6	1.2		.60	2.5	1.2	
Fruits and vegetables		N = 150		.86		N = 153		.84
When you are too tired to make them	.88	3.2	1.2		.82	3.2	1.2	
When your store doesn’t have a good selection	.87	3.1	1.2		.66	3.2	1.2	
When your family wants to eat junk food instead of fruits and vegetables	.85	3.3	1.3		.89	3.5	1.2	
When you are eating out at a restaurant	.77	3.0	1.2		.63	3.1	1.3	
Sugary drinks		N = 147		.88		N = 152		.87
When other members of my house drink it	.89	3.6	1.4		.82	3.7	1.4	
When it’s cheap to buy	.87	3.7	1.4		.79	3.7	1.4	
When we eat at a restaurant	.84	3.2	1.5		.68	3.3	1.5	
When everyone else in the neighborhood drinks it	.84	3.7	1.4		.90	3.8	1.4	
Fruit juice		N = 158		.84		N = 142		.86
When other family members demand it	.87	3.5	1.4		.85	3.4	1.4	
When I feel tired	.85	3.2	1.4		.86	3.0	1.4	
When other members of my house drink it	.84	3.4	1.3		.73	3.1	1.3	
When my child won’t drink milk	.73	3.4	1.4		.68	3.2	1.4	

**Table 3:** Confirmatory factor analysis for self-efficacy for four behaviors

				**Fit indexes**				
**Behavior**	**χ2**	***df***	***p***	**CFI**	**SRMR**	**RMSEA**	**RMSEA 90% CI**	**Alpha**
Physical activity	.85	2	.66	1.00	.01	.00	.00 – .12	.80
Fruits & vegetables	1.86	2	.40	1.00	.02	.00	.00 – .16	.84
Sugary drinks*	7.82	2	.02	.99	.02	.07	.00 – .19	.87
Fruit juice	2.18	2	.34	1.00	.02	.03	.00 – .17	.86

Note. *Least squares method (Normal Distribution Theory); χ2 = Chi-Square; *CFI*: Comparative fit index; *RMSEA*: Root mean squared error of approximation; *SRMR*: Standardized Root Mean Residual.

**Table 4:** Test-retest reliability for one week and two week samples

**Behavior**	**One week test-retest**						**Two week test-retest**					
	**n**	**Mean (SD)**		**Mean (SD)**		**ICC**	**n**	**Mean (SD)**		**Mean (SD)**		**ICC**
		**Score 1**		**Score 2**				**Score 1**		**Score 2**		
Physical activity	107	11.47	(4.17)	11.85	(4.35)	.86	37	11.92	(4.13)	12.46	(4.05)	.84
Fruits & vegetables	107	12.98	(4.07)	13.48	(3.91)	.80	37	12.76	(4.57)	13.32	(3.77)	.80
Sugary drinks	104	14.63	(4.60)	14.97	(4.40)	.88	36	14.36	(4.43)	13.64	(4.73)	.56
Fruit juice	105	13.25	(4.59)	13.56	(4.72)	.84	35	13.27	(4.68)	12.91	(4.74)	.71

Note: *ICC*: Intraclass correlation coefficient.

The construct validity was assessed with Spearman rank correlations because the data for the behavioral measures were non-normal. Correlations were significant in the expected direction for all behaviors ranging from .13 - .29.

#### Sugary drinks

Self-efficacy for sugary drinks was correlated with summary consumption scores from the Block questionnaire, total sugary beverage frequency and total sugary beverage calories (rs = -.29, p < .001). Analysis of variances comparing self-efficacy scores across the response categories for typical amounts of regular soda (F (3, 297) = 11.57, p < .001), fruit drink (F (5, 291) = 9.90, p < .001.), and water (F (4, 293) = 2.74, p < .03) were statistically significant. Pairwise comparisons showed that those parents who reported no regular soda had significantly higher self-efficacy than parents who reported either 12 ounces a day or 24 ounces a day (p < .009) of soda. Parents who reported no fruit drink consumption by their child had significantly higher self-efficacy compared to every other response category (p < .008) of fruit drink, and those who reported that their child drinks more than three glasses of water on a typical day had higher self-efficacy than those parents who reported their child drinks “less than 8 ounces,” (p < .02).

#### 100% fruit juice

Correlations with the Block scores were (*r*s = -.14, p < .03) for sugary beverage frequency and total sugary beverage calorie. Analysis of variances comparing self-efficacy scores across the response categories for typical amounts of water consumed was significant (F (3, 297) = 4.79, p < .002) with Tukey post hoc tests showing differences between those who reported more than 3 glasses and those who reported less than 1 glass (p < .02) and those who reported about 2 glasses (p <. 007). However, the typical amounts of 100% fruit juice was not significant (F (5, 288) = 2.07, p < .07).

#### Fruit and vegetables

Correlations with the fruit and vegetable scores from the Block, i.e., cup equivalents, and the self-efficacy summary score were significant for the total fruit and vegetable cup equivalents (r = .13, p < .03) and vegetables only not including potatoes (r = .15, p < .02) but not for fruit only (r = .10, p > .09).

#### Physical activity

Self-efficacy scores were correlated with the total minutes of physical activity in the past seven days (r = .22, p < .001) and total MET hours (r = .21, p < .002). Self-efficacy was also correlated with total minutes reported playing outside (r = .15, p < .03).

## Discussion

We developed and evaluated the reliability and validity of parent self-efficacy measures for four recommended behaviors for obesity prevention. These scales are novel in that previous efforts have not used sequential methods of measure development, have not adequately covered the domain of barrier self-efficacy and have not conceptualized parent’s confidence in their ability to support their child’s efforts in lifestyle behaviors recommended for the prevention of obesity. Lastly, these scales performed as expected across levels of healthy and unhealthy behaviors providing preliminary evidence of construct validity.

The development and evaluation of these scales provides researchers with new tools that may be useful in understanding more about parent self-efficacy for these four behaviors and how self-efficacy is related to successful weight maintenance and its role as a mediator of behavior change. In addition, the scales may be useful for pediatric providers who would like to screen certain parental behaviors and skills prior to patient/parent counseling. Pediatric providers who use motivation interviewing techniques [28,42] that include the “confidence ladder,” (i.e., a single question to gauge the patient’s confidence in engaging in the health behavior followed by asking the patient what needs to change in order to increase one’s confidence) could use these scales to supplement their counseling efforts. Each scale contains four items, brief enough to be tracked onto intake forms or electronic health records yet long enough to adequately capture the construct [20,21] of parent’s self-efficacy in helping or encouraging healthy changes.

With the growing use of electronic health records (EHR) and a commitment to “meaningful use”, clinicians, EHR vendors, and national quality standards developers will likely look for reliable and valid measures that can be included in EHRs and are brief enough to be used in busy primary care settings. In addition, an increasing number of EHRs are directly linked to patient-portals and mobile devices that can engage patients outside clinical settings for more extensive assessment and counseling activities. Future implementations of these electronic systems could facilitate decision support that uses data collected in primary care settings to support tailored clinical assessment and counseling about self-efficacy and other behavioral factors. For example, they could combine assessment of current health behavior with evaluation of confidence and/or self-efficacy to tailor interview content and goals selected. The scales described in this paper were also developed keeping in the mind the need for tracking health behaviors across time, a function that is made much easier within EHRs.

The utility of these scales should be compared to the shorter confidence ladder (0 to 10 scale, how confident are you that you could drink no sugary drinks?). While the ladder is meant to be administered by a provider during the visit and the scales in this study are meant to be administered prior to the visit (computer, paper), the two methods could be compared to assess their feasibility in primary care settings and to better understand what method results in better patient care process and outcomes. Tenets of measurement theory posit that a four item scale should capture self-efficacy more accurately than a one item question. Additionally, asking the parent these questions prior to the visit may result in a more truthful response than during a provider visit.

### Limitations

The findings of this study are limited because it is not known if the sample used in this study generalizes to other population groups. Although this convenience sample used was from a fairly diverse urban population, it was not large enough for subsample comparisons. Family structure and number of children may influence needs and barriers, e.g., single parents, two parents, extended families, one child, and multiple children. Additionally, parents of children of all ages should be examined to understand how child age influences parent’s self-efficacy. Future studies should seek to recruit a representative sample and examine the extent to which these scales generalize across populations using invariance testing methods. Another caveat is the limitations of using self-report in the creation of the self-efficacy scales and the measures used to validate these scales. Objective measures, such as direct observation of a parent’s behaviors and interactions with the child, would provide better evidence of the scales validity. Parents reported on the child’s diet, physical activity and recreational time yet often they were not caring for their children during the day (e.g., while at work) and may not be very knowledgeable about what happens during these times. However, this type of measurement error should result in a type II error.

## Conclusion

Four scales were developed to assess parental self-efficacy in their ability to support their child’s efforts in avoiding sugary drinks, limiting 100% fruit juice to four to six ounces, consuming recommended amounts of fruits and vegetables, and participating in at least one hour of physical activity every day. The psychometric qualities of each scale met criterion for good reliability and validity. Future studies are necessary to determine the generalizability of these scales to sub-populations that frequent pediatric primary care and the actual use of them in research and clinical practice.

## Abbreviations

SE: Self-efficacy; CFI: Comparative fit index; RMSEA: Root mean squared error of approximation; SRMR: Standardized Root Mean Residual; ICC: Intraclass correlation coefficient.

## Competing interests

JAW, WGA, RL, DB, and RHF have nothing to disclose.

## Authors’ contributions

Study was funded by the National Cancer Institute, K07CA113643. The authors’ contributions: JAW conceived and carried out the study design, secured funding, data collection, data analysis, data interpretation, literature search, generation of the figures and tables, and writing of the manuscript. WGA assisted with the study design and concept, secured funding and reviewed the manuscript. Donna Berry critically reviewed and contributed to the manuscript. Robert LaForge provided statistical consultation, reviewed and contributed to the data analysis and interpretation and critically reviewed and contributed to the manuscript. Robert H. Friedman conceived the study concept and design, assisted in securing funding and critically reviewed and contributed to the manuscript. All authors had final approval of the submitted and published versions.
